# The impact of a community driven mass media campaign on the utilisation of maternal health care services in rural Malawi

**DOI:** 10.1186/s12884-016-0816-0

**Published:** 2016-01-27

**Authors:** Collins O. F. Zamawe, Masford Banda, Albert N. Dube

**Affiliations:** Parent and Child Health Initiative (PACHI), Research Centre, Amina house, Off Chilambula Road, P.O. Box 31686, Capital City, Lilongwe, 3 Malawi; The Ministry of Health, Lilongwe, Malawi; MaiMwana Project, P.O. Box 2, Mchinji, Malawi; The University of Malawi, College of Medicine, School of Public Health, Blantyre, Malawi

**Keywords:** Women’s health, Community intervention, Radio campaign, Phukusi la Moyo

## Abstract

**Background:**

Mass media is critical in disseminating public health information, improving health knowledge and changing health behaviours. However, most of the mass media public health interventions do not sufficiently engage the local people; they are externally determined. Due to this, very little is known about the effects of locally instigated mass media promotion. Therefore, the aim of this study was to examine the impact of a community driven mass media campaign called Phukusi la Moyo (tips of life) on the utilisation of maternal health care services.

**Methods:**

A community-based cross-sectional study involving 3825 women of reproductive age (15–49 years) was conducted in rural Malawi to evaluate the Phukusi la Moyo (PLM) campaign. To do this, we compared the utilisation of maternal health care services between women who were exposed to the PLM campaign and those who were not. Respondents were identified using a multistage cluster sampling method. This involved systematically selecting communities (clusters), households and respondents. Associations were examined using Pearson chi square test and a multivariable logistic regression model.

**Results:**

The likelihood of using contraceptives (AOR = 1.61; 95 % CI = 1.32–1.96), sleeping under mosquito bed-nets (AOR = 1.65; 95 % CI = 1.39–1.97), utilising antenatal care services (AOR = 2.62; 95 % CI = 1.45–4.73) and utilising postnatal care services (AOR = 1.59; CI = 1.29–1.95) were significantly higher among women who had exposure to the PLM campaign than those who did not. No significant association was found between health facility delivery and exposure to the PLM campaign.

**Conclusion:**

Women exposed to a community driven mass media campaign in rural Malawi were more likely to utilise maternal health care services than their unexposed counterparts. Since, the use of maternal health care services reduces the risk of maternal morbidity and mortality, community-led mass media could play a significant role towards improving maternal health outcomes in low-and-middle-income countries. Therefore, we recommend the use of locally driven mass media in disseminating public health information in limited resource settings.

## Background

Many people needlessly die every day due to lack of access to health information which would have allowed them or health workers to make appropriate decisions [1]. Thus, access to reliable health information is a cornerstone of improved and sustainable health outcomes [2]. In low-and-middle-income countries (LMIC), poor access to health information, especially among rural women is a major public health problem [3, 4]. It undermines efforts to bring health care services closer to the people. Hence, innovative strategies are required to meet the goal of ‘Health Information For All’ (HIFA) campaign [1].

Mass media is one of the popular and cost-effective public health promotion tools globally [2, 5, 6]. It is the main source of health information in many parts of the world, especially in LMIC. In Nigeria, for example, mass media is one of the major sources of information about malaria [7], women’s health, HIV/AIDS and family planning among others [3]. Likewise, access to information in rural Tanzania is mainly through mass media (radio, television, newspapers), cell-phones and face to face communication [5]. Moreover, mass media interventions have been at the centre of maternal health and HIV prevention in Malawi [4, 8].

A growing body of evidence shows that mass media impacts public health knowledge, attitudes, beliefs and behaviours [9–11]. In Zambia, for example, exposure to radio and television programs about condom and HIV/AIDS was associated with higher condom use [12]. Similarly, a behaviour change communication media campaign in Uganda improved knowledge of six months as the ideal period for exclusive breastfeeding [13]. In addition, media exposure significantly improved dietary diversity and meal frequency among infants and children in Ethiopia [14]. Further, a host of HIV media campaigns implemented between 1998 and 2007 in 23 countries effectively stimulated behaviour change [15].

In particular, the effects of mass media on maternal health behaviours have long been recognised in LMIC, where most of the maternal deaths occur [6, 16, 17]. In Nepal, exposure to mass media increased utilization of antenatal care services among rural women [18]. Comparable observations were made in Ghana, where exposure to mass media was recognized as a significant predictor of the likelihood that a woman would utilize antenatal care services and opt for childbirth assisted by a skilled provider [19]. Moreover, mass media impact men’s involvement in maternal health. For example, Malawian women exposed to a specific media campaign reported higher participation of men in antenatal, postnatal and delivery care services [20].

Evidently, mass media is critical in disseminating public health information, improving health knowledge and changing health behaviours. Nonetheless, mass media campaigns have had inconsistent outcomes [3, 21]. A review of media campaigns on contraceptive use in LMIC demonstrated that most of the interventions had short-term effects, to the extent that demand for contraceptives would fall back to the initial level [22]. At country level, radio communication campaigns in Malawi had limited effect on condom use [4]. Likewise, media campaigns in Bangladesh appear to have no impact on contraceptive use [23].

Unreliable outcomes of mass media interventions in public health could be down to three factors: (a) media messages do not address cultural and practical barriers to behaviour change, (b) use of inappropriate media platforms to reach rural target audiences and (c) programs focus on one issue in the face of other related problems [24]. In short, most of the mass media interventions do not sufficiently engage the local people or community members [6, 25]. Usually, what is branded as ‘community-based mass media’ merely specifies that the “unit of intervention is at the community level” [3, 6, 7, 18]. To be truly community grounded, an intervention is supposed to be developed and spearheaded by and for the communities it aims to serve [6]. Such interventions are more likely to be acceptable, sustainable and cost-effective [26–28].

Lack of community involvement implies that most of the public health mass media interventions are externally determined [6, 29, 30]. Owing to this, very little is known about the effects of locally instigated mass media promotion. For this reason, the aim of this study was to examine the impact of a mass media campaign (Phukusi la Moyo), which was initiated and led by the community members, on the utilisation of maternal health care services. The study will provide critical insights into the role of the community members in public health mass media interventions. This would help to inform and strengthen designs of future media interventions in maternal health and other global health areas.

## Methods

### A synopsis of the Phukusi la Moyo campaign

The Malawi Ministry of Health in collaboration with the Institute for Global Health (University College London) established a project called MaiMwana (mother and child) in 2003. The aim of the project was “to build capacities of communities in rural Malawi to take control of mother and child health issues that affect them” [31, 32]. The project was set up in Mchinji district, which was divided into 48 clusters. Of these, 24 received the women’s group intervention [31]. Women in treatment clusters formed 207 groups to facilitate learning, sharing of lessons and to provide a platform for dealing with maternal health problems locally. The intervention was divided into four phases, with each having its main objective as follows: (a) to identify priority problems affecting women during pregnancy, delivery and postpartum; (b) to plan for the solutions of the problems identified and prioritised; (c) to implement the locally driven solutions to the problems and (d) to evaluate the solutions [31].

One of the major priority problems that most of the groups identified during phase one of the project was that health information was not accessible in some parts of the district due to shortage of community health workers. As a result, during phase two, the groups identified the feasibility of using a community radio to bring knowledge about maternal and child health issues to a large population. The groups agreed to lobby for a maternal health education program to be aired on the district’s community radio station called “Mudziwanthu” (our community). Following that, a weekly radio program called “Phukusi la Moyo” (tips of life) was born. The intervention was implemented between 2009 and 2011.

As a radio campaign, the focus of Phukusi la Moyo (PLM) was to provide comprehensive maternal health information to women of reproductive age (15–49 years). The ultimate goal of the PLM campaign was to help reduce maternal and child mortality through improving utilisation of maternal health care services [31, 32]. The PLM’s emphasis was on raising awareness of the risks associated with pregnancy, the importance of antenatal and postnatal care, and the advantages of delivering at the health facility. The campaign also aimed to promote (a) men’s engagement in maternal health, (b) the use of mosquito bed-nets and (c) uptake of malaria prophylaxis among pregnant women.

The PLM campaign messages were delivered by means of different activities. These included panel discussions, community discussions, drama and songs. Members of the women’s groups developed the radio programs with the technical support of the MaiMwana project and the Mudziwanthu radio station. The communities formed radio listening clubs, where women could listen to the program in groups and discuss the key messages afterwards.

### The study design

A community-based cross-sectional study was conducted to evaluate the PLM interventions. This involved comparing the utilisation of maternal health care services between women who were exposed to the PLM campaign and those who were not. In the absence of the baseline data, this design was considered appropriate for the evaluation.

### The study setting

The study was implemented in Mchinji, one of the 28 districts in Malawi. It is a typical rural district located in the central region of Malawi. Mchinji was particularly chosen because it was the catchment area of the PLM campaign, which this study intended to evaluate. The MaiMwana project divided the district into 48 equal clusters based on population size. This study was conducted in 24 of these 48 clusters, which were selected using a simple random sampling method.

### Population of the study and sample size

Women of reproductive age (15–49 years) who gave birth or fell pregnant at least 12 months after the implementation of the PLM campaign were the target population of this study. The study engaged women only because the objective was to examine the utilisation of maternal health care services. Other inclusion criteria included: stayed continuously in the community between 2009 and 2013, willingness to participate and ability to provide informed consent. To allow meaningful analysis at cluster-level, the study included statistically reasonable number of respondents from each cluster. This was computed using G*Power version 3.1 program [33]. The minimum cluster sample size was 143 (effect size = 0.3; power = 0.80; alpha < 0.05). Overall, data were collected from 3825 women.

### Data collection and sampling methods

The data collection period was from July to December 2013. A team of 14 local research assistants conducted the fieldwork. They received a short training in research ethics and data collection. Data were collected using pretested structured electronic questionnaires, which were put in the Personal Digital Assistants (PDAs). Among other things, the questionnaire captured demographic details, exposure to the PLM program and utilisation of maternal health care services during the last pregnancy.

Respondents were systematically identified through a multistage cluster sampling method. In the first stage, the clusters were chosen using a simple random sampling technique. This was followed by selection of households through a systematic sampling approach. The sampling frame for each cluster was 160. Finally, a single respondent was identified from each household using a simple random sampling method. The study registered 100 % response rate.

### Description of study variables

Five outcome variables were examined in this study. These were (a) utilisation of antenatal care services, (b) use of mosquito bed nets during pregnancy, (c) delivery at the health facility, (d) utilisation of postnatal care services and (e) use of contraceptives. All outcome variables were dichotomous (yes/no). Exposure to the PLM campaign was the main independent variable of this study. Respondents were either exposed or not (no/yes). The following covariates were also measured: highest education level, occupation, marital status and participation in the women’s group intervention.

### Data analysis

The level of missing values in all relevant variables was below 5 %. As a result, all cases with missing values were discarded (*n* = 386) [34]. The final sample size of the study was 3439. Stata (version 12) was used for analysis. Descriptive statistics were used to describe variables. Then, we examined the relationships between independent and outcome variables using the Pearson chi-square (χ^2^) test of independence. Finally, a multivariable logistic regression model was fitted to determine the direction and degree of the associations between the independent and outcome variables. Each outcome variable was examined twice against the independent variable: first, without adjusting for covariates and then, after controlling for marital status, age, occupation, participation in women’s groups and education level. The relationships were estimated using crude and adjusted odds ratios and a 95 % confidence interval. We used alpha (α) of *p* < 0.05 to determine statistical significance.

### Ethical clearance and consent

The protocol of this study was reviewed and approved by Malawi’s National Health Sciences Research Committee (NHSRC). Participation in this study was voluntary and written informed consent was obtained from all the participants. Data collectors explained relevant details of the study to each potential respondent and asked if she would be interested in participating. Participants were required to sign or thumb-stamp the consent form. For participants under 18 years old (minors), additional written consent was obtained from their parents or guardians.

## Results

### Characteristics of the participants

Table 1 presents descriptive and bivariate results. Overall, the study engaged 3439 women residing in Mchinji district. The largest subgroup of the respondents were young women aged between 20 and 29 (47 %) followed by those between 30 and 39 years old (34 %). Very few teenage girls (7 %) and older women over the age of 40 (12 %) were involved. Most of the respondents at least attended primary education (72 %), but none had gone beyond secondary school. A small number of participants (15 %) had no formal education. Most of the participants (74 %) were subsistent farmers. Regarding marital status, most of the respondents were married (90 %). Additionally, many participants were members of the women’s groups (68 %).

**Table 1:** Characteristics of the participants and bivariate results (*n* = 3439)

Variable	n (%)		Exposure to PLM program				*p*-value
			No (%)		Yes (%)		
Total	3439	(100.0)	1349	(39.2)	2090	(60.8)	
Age (years)							
15–19	224	(6.5)	102	(45.5)	122	(54.5)	<0.003*
20–29	1628	(47.3)	590	(36.2)	1038	(63.8)	
30–39	1183	(34.4)	480	(40.6)	703	(59.4)	
40–49	404	(11.8)	177	(43.8)	227	(56.2)	
Education							
None	529	(15.4)	261	(49.3)	268	(50.7)	<0.001*
Primary school	2463	(71.6)	964	(39.1)	1499	(60.9)	
Secondary school	447	(13.0)	124	(27.7)	323	(72.3)	
Marital status							
Never married	99	(2.9)	53	(53.5)	46	(46.5)	<0.001*
Currently married	3094	(90.0)	1180	(38.1)	1, 914	(61.9)	
Formerly married	246	(7.2)	116	(47.1)	130	(52.9)	
Occupation							
Farmers	2559	(74.4)	1033	(40.4)	1526	(59.6)	<0.001*
Self employed	514	(15.0)	159	(30.9)	355	(69.1)	
Casual worker	273	(7.9)	119	(43.6)	154	(56.4)	
Others	93	(2.7)	38	(40.9)	55	(59.1)	
Women’s group member							
No	2341	(68.1)	1090	(46.6)	1251	(53.4)	<0.001*
Yes	1098	(31.9)	259	(23.6)	839	(76.4)	
Used contraceptives							
No	529	(15.4)	267	(50.5)	262	(49.5)	<0.001*
Yes	2910	(84.6)	1082	(37.2)	1828	(62.8)	
Utilised antenatal health care services							
No	54	(1.6)	35	(64.8)	19	(35.2)	<0.001*
Yes	3385	(98.4)	1314	(38.8)	2071	(61.2)	
Sleeping under bed-nets during pregnancy							
No	710	(20.6)	365	(51.4)	345	(48.6)	<0.001*
Yes	2729	(79.4)	984	(36.1)	1745	(63.9)	
Delivered at health facility							
No	271	(7.9)	116	(42.8)	155	(57.2)	0.209**
Yes	3168	(92.1)	1233	(38.9)	1935	(61.1)	
Utilised postnatal health care services							
No	469	(13.6)	233	(49.7)	236	(50.3)	<0.001*
Yes	2970	(86.4)	1116	(37.6)	1854	(62.4)	

*PLM* phukusi la moyo

*Significant (*p* < 0.05); **Not significant (*p* > 0.05)

### Sources of maternal health information and use of maternal health services

**Fig. 1:**
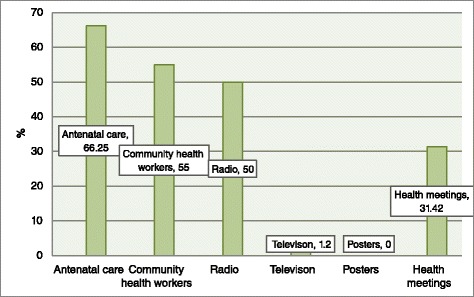
Sources of maternal health information (multiple responses)

Different sources of maternal health information were reported by our respondents. Most of them mentioned antenatal clinics followed by community health workers and radio (Fig. 1). Moreover, most participants (70 %) acknowledged that they had access to the radio. In particular, around 60 % of the participants were exposed to the PLM campaign (Table 1). We also assessed utilisation of maternal health care services. Women were asked about utilisation of maternal health care services during their last pregnancy. Almost all the women (98.4) attended antenatal care clinics, even though it was not certain if they fulfilled the recommended minimum of four visits. Similarly, most of the participants used contraceptives, delivered at the health facility, used postnatal care, and were sleeping under mosquito bed-net during pregnancy.

### Impact of the PLM campaign

We examined the association between exposure to the PLM campaign and the utilisation of maternal health care services. The results indicate that there was a significant association between exposure to the PLM campaign and use of contraceptives (*p* ≤ 0.001), utilisation of antenatal care (*p* ≤ 0.001), sleeping under mosquito bed-nets (*p* ≤ 0.001) as well as utilisation of postnatal care (*p* ≤ 0.001). No significant association was found between exposure to the PLM program and delivery at the health facility (*p* = 0.209).

The results further show that the likelihood of using contraceptives (OR = 1.72; 95 % CI = 1.43–2.07), mosquito bed-nets (OR = 1.88; 95 % CI = 1.59–2.22), antenatal care services (OR = 2.90; 95 % CI = 1.65–5.10), and postnatal care services (OR = 1.64; CI = 1.35–1.99) were significantly higher among women who had exposure to the PLM campaign than those who did not. After adjusting for potential confounders (i.e. age, education, occupation, marital status, participation women’s groups), women who had exposure to the PLM campaign were still significantly more likely to use contraceptives (AOR = 1.61; 95 % CI = 1.32–1.96), mosquito bed-nets (AOR = 1.65; 95 % CI = 1.39–1.97), antenatal care services (AOR = 2.62; 95 % CI = 1.45–4.73), and postnatal care services (AOR = 1.59; CI = 1.29–1.95) than those who were not exposed; although with some reductions (Table 2). No significant association was found between health facility delivery and exposure to the PLM campaign.

**Table 2:** Exposure to the PLM campaign and the utilisation of maternal health care services (logistic model) (*n* = 3439)

Outcome variables	OR	(95 % CI)	*p*-value	AOR	(95 % CI)	*p*-value
Used contraceptives						
No (Rc)	1.00					
Yes	1.72	(1.43–2.07)	<0.001*	1.61	(1.32–1.96)	<0.001*
Utilised antenatal care services						
No (Rc)	1.00					
Yes	2.90	(1.65–5.10)	<0.001*	2.62	(1.45–4.73)	0.001
Slept under bed-net						
No (Rc)	1.00					
Yes	1.88	(1.59–2.22)	<0.001*	1.65	(1.39–1.97)	<0.001*
Delivered at a health facility						
No (Rc)	1.00					
Yes	1.17	(0.91–1.51)	0.209**	1.11	(0.85–145)	0.44**
Utilised postnatal care services						
No (Rc)	1.00					
Yes	1.64	(1.35–1.99)	<0.001*	1.59	(1.29–1.95)	<0.001*

*OR* Odds ratio

*AOR* Adjusted odds ratio

*CI* Confidence interval

*Rc* Reference category

*Significant (*p* < 0.05)

**Not significant (*p* > 0.05)

## Discussion

A community driven mass media campaign in rural Malawi was associated with improved utilisation of maternal health care services among women who were exposed to it. Specifically, exposure to the PLM campaign increased the likelihood of using contraceptives by 61 %, utilising antenatal care services by 162 %, sleeping under mosquito bed nets during pregnancy by 65 % and utilising postnatal care by 59 %. This suggests that community driven mass media interventions are effective in improving uptake of maternal health care services in limited resource settings.

The main focus of PLM was to raise awareness of the maternal health complications and the importance of family planning, antenatal care, postnatal care and delivering at the health facility. Therefore, increased utilisation of maternal health care services among women exposed to the PLM campaign was likely due to improved knowledge of maternal health issues. This is consistent with other studies in LMIC that have shown that mass media campaigns influence knowledge and behaviours [35, 36]. However, most of the earlier mass media promotions were externally developed with no input from the targeted communities [6, 8, 18]. The PLM campaign was different; it employed a bottom-up approach. Therefore, the results of this study add a new dimension to the literature of mass media in global health.

Moreover, our outcomes demonstrate that a true community centred approach is conceivable in mass media. Like other health programs, we believe that the benefits of mass media would be maximised if the campaigns are grounded in the targeted communities. For instance, apart from the intended outcomes, community involvement in public health mass media interventions would probably promote self-reliance/ownership, encourage the use of locally available resources and address priority health needs [37, 38]. To this effect, where appropriate, a bottom-up approach should be prioritised in media interventions targeting the rural masses. In the meantime, however, there is a need to compare the impact of the community driven and externally determined media interventions.

In particular, community driven media campaigns could be critical in improving maternal health outcomes in LMIC. Although the utilisation of maternal health care services reduces the risk of maternal morbidity and mortality, some of the women continue to shun these health care services [17, 39, 40]. The findings of this research reveal that locally driven mass media could be a reliable instrument for enhancing uptake of maternal health care services.

The findings of this study further indicate that mass media is or could be an essential platform for transmitting health information in rural areas. Over 70 % of the participants had access to the radio and about 50 % of the women also indicated that the radio was the main source of maternal health information. Similar findings have also been observed elsewhere [3, 5, 41]. Since the shortage of community health workers makes it impossible to reach every woman (especially in rural areas) with maternal health information [42], community driven mass media campaigns could be a reliable alternative.

While recommending community driven mass media campaigns, we would like to recognise the need for the community members to adequately engage professional health care workers in the production of campaign materials. This would ensure that correct information is made available. Besides, more community radio stations should be set up in rural places. This would allow broadcasting of tailor-made programs like the PLM that meet the specific needs of the people in a particular setting. Equally, it is logical to expand the signal radius of the existing community radio stations to reach out to more people living in the remote areas.

This study had some limitations and strengths. Due to the cross-sectional design, we could not establish causal-effect relationships. Besides, owing to the self-reporting of both exposure and health behaviours, we cannot rule out the recall and social desirability biases. Nonetheless, the statistical significance was robust due to relatively large sample size.

## Conclusion

Women exposed to a community driven mass media campaign in rural Malawi were significantly more likely to utilise maternal health care services than their unexposed counterparts. Since, the use of maternal health care services reduces the risk of maternal morbidity and mortality, community driven mass media could play a significant role towards improving maternal health outcomes in LMIC. To this end, community driven mass media interventions should be promoted, especially in limited resource settings.

## Abbreviations

AOR: adjusted odds ratio
CI: confidence interval
LMIC: low-and-middle-income-countries
NHSRC: National Health Sciences Research Committee
PDA: Personal Digital Assistants
PLM: Phukusi la Moyo
